# The Postictal Phase in Canine Idiopathic Epilepsy: Semiology, Management, and Impact on the Quality of Life from the Owners’ Perspective

**DOI:** 10.3390/ani14010103

**Published:** 2023-12-27

**Authors:** Charlotte Kähn, Nina Meyerhoff, Sebastian Meller, Jasmin N. Nessler, Holger A. Volk, Marios Charalambous

**Affiliations:** Department of Small Animal Medicine and Surgery, University of Veterinary Medicine Hannover, 30559 Hannover, Germany

**Keywords:** veterinary neurology, epilepsy, canine (dog), postictal, quality of life, impact

## Abstract

**Simple Summary:**

This survey is the first study assessing the semiology and treatment approach of the postictal phase in dogs with idiopathic epilepsy as well as its impact on the quality of life of both dogs and their owners. The postictal phase has been minimally studied in both human and veterinary medicine. This study aimed to understand postictal semiology, assess its impact on the quality of life, highlight its importance in managing seizure disorders, and explore potential therapeutic approaches for postictal symptom management. The outcomes of this research bring attention to the deficiencies in epilepsy treatment and indicate avenues for additional research that can enhance the quality of life.

**Abstract:**

Background: Dogs with idiopathic epilepsy experience not only the preictal and ictal seizure phases but also the postictal phase. To date, research has primarily focused on the preictal and ictal semiology and therapeutic control of ictal events. Research into the postictal phase‘s pathophysiology, as a therapeutic target and how it impacts the quality of life, is sparse across different species. Interestingly, even if anecdotally, owners report the postictal period being an impactful negative factor on their quality of life as well as their dog’s quality of life. Hypothesis/objectives: We aimed to assess the semiology and the impact of postictal signs on the quality of life of owners and dogs. Method: This observational study was carried out using surveys of owners of dogs with seizure disorders. Results: The questionnaire was filled out by 432 dog owners, 292 of whom provided complete responses that could be analysed. More than nine out of ten owners (97%) reported the presence of various postictal clinical signs. The dog’s and the owner’s quality of life was mainly affected by specific postictal signs, i.e., disorientation (dog: 31%; owner: 20%), compulsive walking (dog: 17%; owner: 22%), ataxia (dog: 12%; owner: 6%), and blindness (dog: 17%; owner: 10%). Nearly 61% of the owners felt that the severity of postictal signs was moderate or severe. Rescue antiseizure medications did not have an effect on controlling the postictal signs based on 71% of the responders. In contrast, 77% of the respondents reported that other measures such as rest, physical closeness, and a quiet and dark environment had a positive impact on the postictal phase. Conclusions and clinical importance: Overall, this survey shows that specific postictal signs are common and have a notable impact on the perceived quality of life of both dogs and their owners. According to the respondents, antiseizure medication might have no influence on the postictal phase in most cases, in contrast to other nonpharmacological measures. Further research on the management of the postictal phase is vital for improving the quality of life of dogs with seizure disorders and their owners.

## 1. Introduction

In veterinary practice, canine epilepsy is one of the most common chronic neurological diseases, with a high prevalence in certain breeds [1,2,3]. Approximately 2.6% of animals presented at clinics exhibit clinical signs of epileptic seizures [4,5]. Certain dog breeds are more affected than others, as shown in a study of over 80,000 dogs in the UK, where Labrador Retrievers, followed by Staffordshire Bull Terriers and Jack Russell Terriers, were the most commonly affected [2]. This complex brain disorder involves recurrent, excessive, and synchronised electrical brain activities that can result in a variety of motor, autonomic, or behavioural changes [6].

The course of a typical epileptic seizure in dogs can be broken down into three distinct phases. An epileptic seizure often starts with a preictal phase, which may not be always noticed by the owner, followed by the ictal phase. Finally, there is the postictal phase, which, despite its importance, has been relatively underexplored in both human and veterinary medicine. There are only a few studies in human and veterinary medicine characterising the postictal phase. The ictus, followed by the postictal phase, exhibits transient central nervous system abnormalities after the disappearance of clinical signs. In humans, this phase is characterised by neurological or psychiatric symptoms, often slowed or suppressed EEG activity, and a duration ranging from minutes to several days [7]. During the postictal phase, neuronal excitability decreases due to inhibitory signals, involving calcium-activated potassium channels, GABA receptors, and other receptors/ion channels [8]. Secondary changes can impair brain function. Studies with rodents have shown persistent hypoxia in brain regions following epileptic seizures, caused by vasoconstriction resulting in reduced blood flow and oxygen deprivation in critical brain areas. Normal brain function gradually recovers [9].

According to a study in human medicine, various neurological, cognitive, and behavioural changes can occur during the postictal phase that can also significantly affect the quality of life (QoL) [7]. So far, few studies have examined psychiatric comorbidities in dogs with idiopathic epilepsy. In one study, 71% of the examined dogs exhibited at least one change in behaviour since the onset of idiopathic epilepsy. These alterations encompass excessive fear/anxiety, abnormal perception (such as barking without apparent cause), abnormal reactivity, attachment disorder, demented behaviour, apathetic behaviour, and aggression [10,11]. Based on a comprehensive veterinary study conducted on dogs, a significant majority of canine epilepsy patients experience a discernible postictal phase following their epileptic seizures [12]. This postictal phase is particularly characterised by a multitude of distinctive and often unsettling clinical signs. In people, the aforementioned disturbances can last minutes to days following the ictal phase and, thus, can lead to disturbance in the regular lifestyle [8]. When comparing the duration of the different phases of seizure activity, it becomes evident that the postictal phase’s extended duration can have a more profound impact on the overall QoL compared to the other phases. In medical interviews in human medicine, the postictal phase is often described as “the worst” part of the disease [13]. 

The main priority in the management of animals with seizure disorders is to treat and prevent the ictal activity [14]. However, this should not be the only goal [15]. Improving the QoL of owners and dogs with epilepsy through various interventions implemented at different stages is of pivotal importance [10], especially since seizures cannot always be adequately controlled and are not the only factor impacting the QoL [16]. In the realm of animal science, it is widely acknowledged that animals possess sentience, signifying their capacity for physical sensation and emotional experiences [17,18,19]. The concept of QoL is ethically valuable, and it is our responsibility as scientists and clinicians to focus on enhancing the future QoL of animals. Epilepsy and all its stages, particularly the long-lasting postictal phase, could have a negative impact on the QoL. 

Neurological conditions often manifest in clinical signs that significantly impact the caregiver. These signs encompass not only epileptic seizures but also clinical signs related to medication side effects and the postictal period, such as increased urination or noticeable changes in mood like appearing depressed or anxious [20]. It is apparent that generalised epileptic seizures can be emotionally distressing for owners to witness [21]. Interestingly, the distress experienced by the owner during seizures may surpass that of the animal, as the animal is unconscious during the event. However, after regaining consciousness, the postictal period could potentially be distressing for both the owner and the animal, especially if the signs persist for an extended duration. Looking after chronically ill animals, owners might grapple with feelings of helplessness [22]. Owners of dogs with epilepsy frequently express enduring feelings of fear, stress, and uncertainty regarding their dog’s health [23]. Owners who have a dog with epilepsy do alter their lifestyle significantly to accommodate their dog’s care needs [23]. This lack of understanding from their friends, family, or colleagues, coupled with the fear of leaving the dog unattended due to this condition, can have notable social implications for some owners [22]. 

Aims of epilepsy management should include not only the treatment of seizure frequency and severity but also an improvement in comorbidities and a reduction in adverse effects, as well as thorough owner education [10,24]. As postictal signs do affect the QoL in people with epilepsy, it is likely that this is also true for dogs with epilepsy and their owners, providing another therapeutic target for improving the QoL. The aim of the current study was to (i) assess the postictal semiology, (ii) evaluate the impact of the postictal phase on the QoL of both dogs and their owners, and (iii) analyse the strategies used by the owners to alleviate the postictal signs in their dogs. By systematically examining the postictal semiology, we aimed to provide a deeper understanding of the varied and often complex array of behaviours and clinical signs exhibited by dogs during this crucial phase, shedding light on the less explored aspects of canine epilepsy.

By identifying novel approaches and interventions, veterinarians could enhance the overall well-being of dogs with epilepsy and alleviate the distress experienced during the postictal phase. This research represents a step towards more holistic and compassionate care for both canine patients and their devoted owners, ultimately improving their QoL and strengthening the human–animal relationship.

## 2. Materials and Methods

An observational study was designed based on surveys from owners of dogs suffering from any type of seizure disorder; however, only those dogs with idiopathic epilepsy were selected for this study. The survey’s questions were developed using veterinary expertise and partially based on a previous questionnaire developed by the authors [25]. The complete survey can be found in Supplementary File S1. The first section consisted of five questions on general information such as the dog’s signalment and general clinical condition. The second section consisted of 16 questions on semiology, course, diagnosis, and treatment of chronic and emergency seizure disorders. The third section included 18 questions on the signs of the postictal phase, how it affects the QoL of the owner and dog, and what measures are chosen to ameliorate the symptoms in the postictal phase. It was possible to view the survey in either English or German. The questionnaire was conducted via the online survey software LimeSurvey (https://www.limesurvey.org/de/; Version 3.23.1+200825; accessed on 1 March 2023) and distributed via social media. To disseminate the survey on social media, the Facebook and Instagram pages of the University of Veterinary Medicine in Hanover were utilised. Additionally, a link was shared in specific groups in which owners with dogs affected by this condition participated. Data were collected from April to June 2023. The questionnaire was published after approval from the university’s data protection officer. Responses were used for analysis when fully completed, and postictal information was available. Descriptive statistics were used to evaluate and present the obtained data. The data were analysed using Microsoft Excel (Version 16.80).

## 3. Results

The questionnaire was filled out by 432 dog owners, 292 of whom provided complete responses for analysis. In addition to incomplete responses, incorrect answers and cases that did not indicate idiopathic epilepsy were excluded. The general information about the study population, including demographic characteristics such as age, sex, and racial distribution, as well as the relevant details about the long-term and emergency seizure medications used in the study population, are summarised in Table 1. 

**Figure 1:**
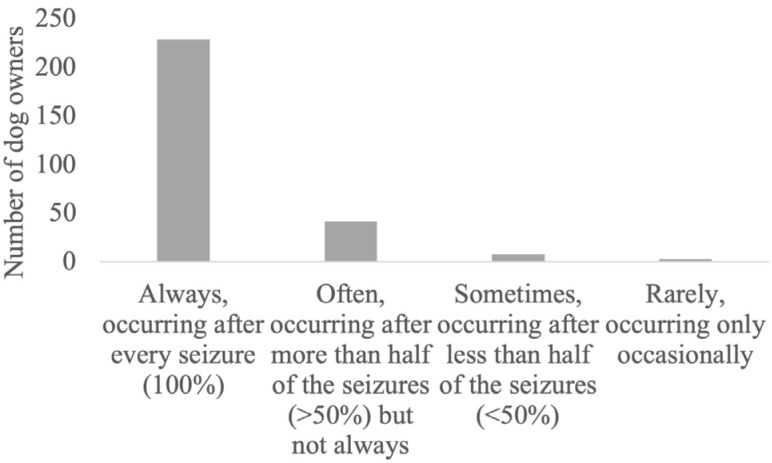
Occurrence of postictal signs after a seizure.

**Figure 2:**
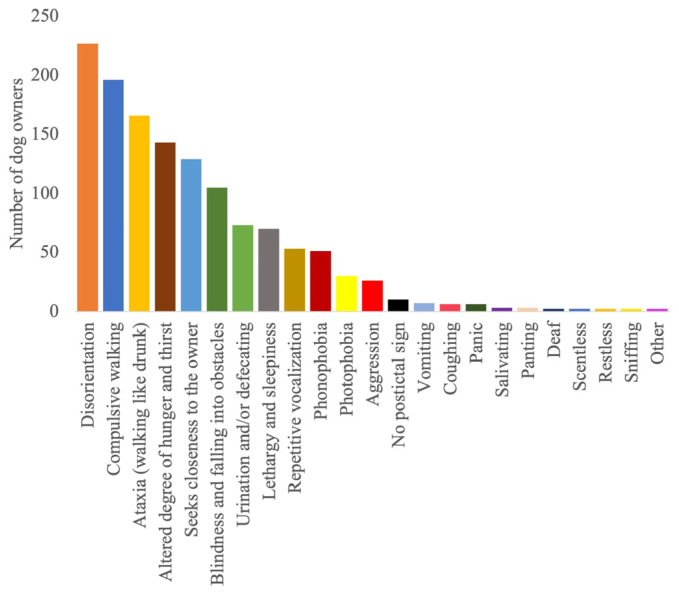
Postictal clinical signs reported by owners of dogs with epilepsy.

**Figure 3:**
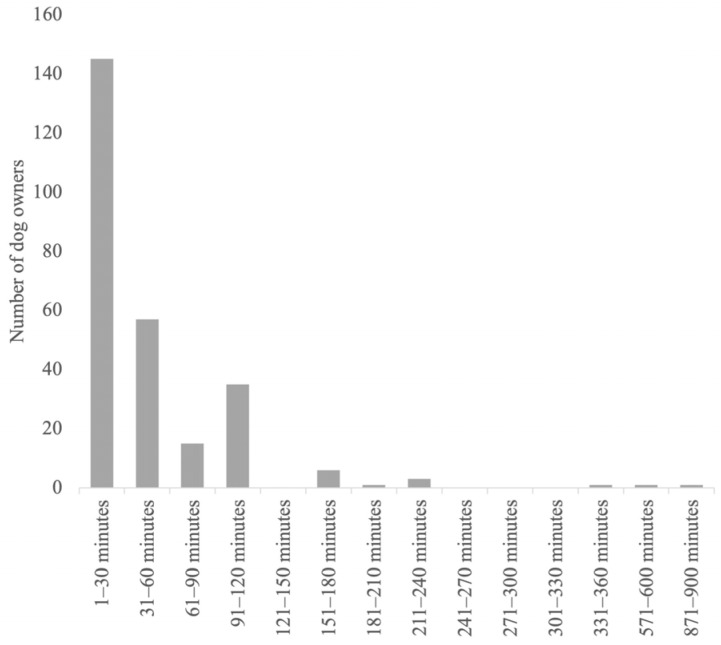
Length of the postictal signs.

**Figure 4:**
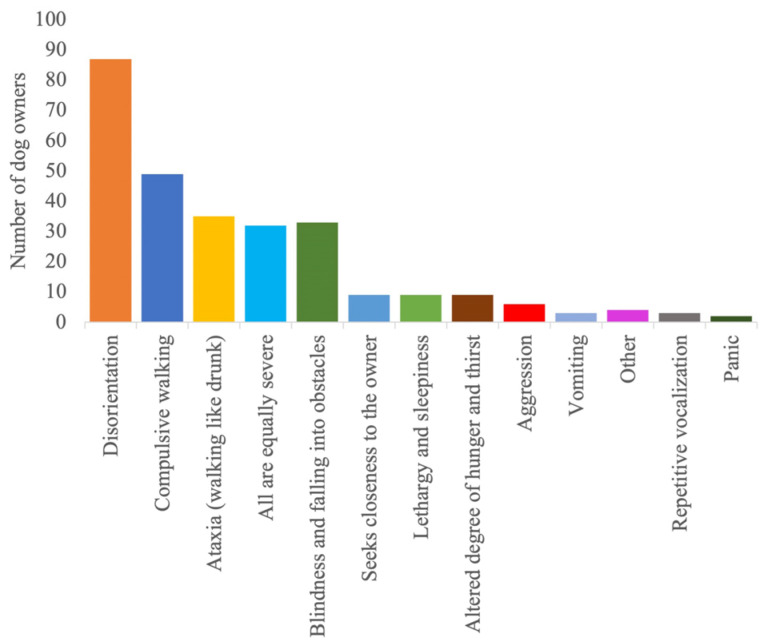
Postictal signs owners reported to most affect their dog’s quality of life.

**Figure 5:**
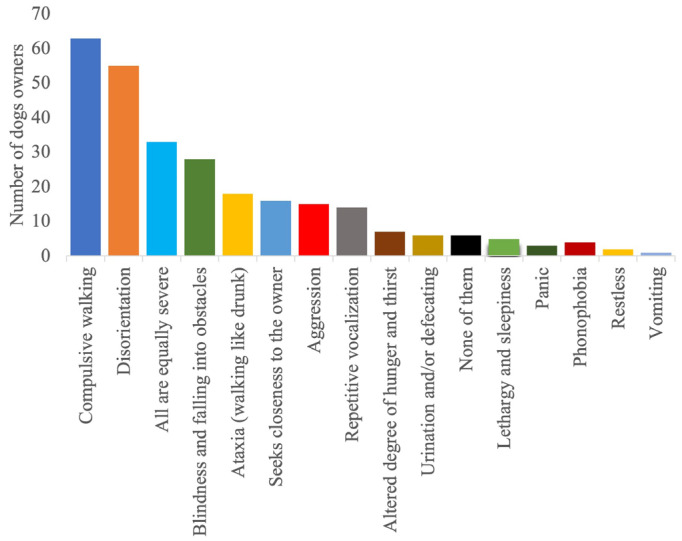
Postictal signs reported that most affect the quality of life of the owner.

**Figure 6:**
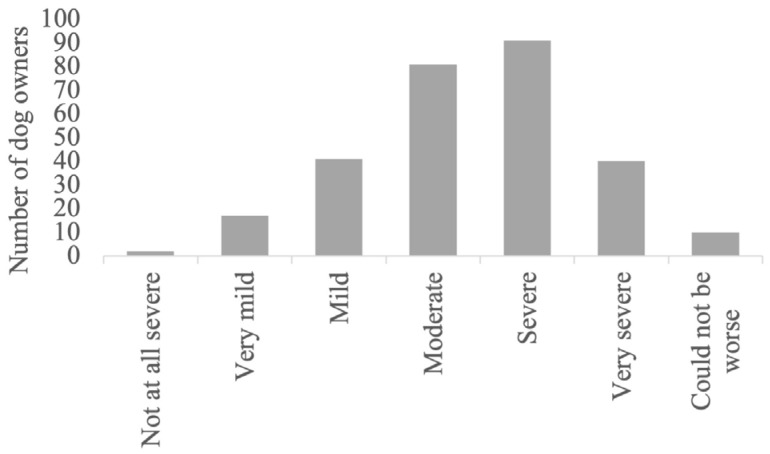
Severity of the postictal signs.

The detailed results regarding the semiology and management of the postictal phase, as well as its impact on the QoL of dogs and owners, are provided in Figure 1, Figure 2, Figure 3, Figure 4, Figure 5 and Figure 6. 

When the postictal phase lasted less than 30 min, 3% of the respondents reported that it reached the highest level of negative impact on their dogs’ QoL. If the postictal phase extended from 31 to 60 min, this percentage remained at 3%. When the postictal phase lasted between 61 and 90 min, 7% of the respondents indicated that the highest degree of negative influence on their dogs’ QoL was reached.

The assessment of the impact on the owners’ QoL revealed the following pattern: In cases where the postictal phase was under 30 min, 3% of the respondents stated that it reached the highest level of negative influence. With a postictal phase lasting between 31 and 60 min, this percentage rose to 7%, and for a duration of 61 to 90 min, it increased to 13%.

Table 2 depicts the owner-reported effects of the administered medication on the postictal phase. 

**Figure 7:**
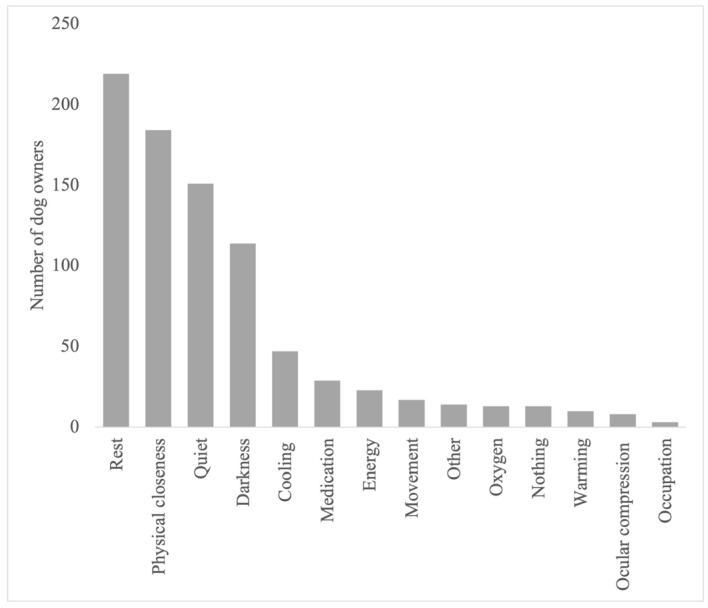
Measures mentioned by owners that could improve the postictal signs.

The most commonly used measures to improve the impact of the postictal phase according to owners were rest (219/282) and calming through physical closeness (184/282), as well as the establishment of a quiet (151/282) and/or dark (114/282) environment; such measures improved the signs according to the opinion of 77% of the owners. Information on the impact of these and less commonly applied other measures is provided in Figure 7.

## 4. Discussion

To our knowledge, this study represents the first comprehensive and targeted investigation into the semiology and treatment strategies employed during the postictal phase in dogs while concurrently shedding light on its profound impact on the QoL for both canine patients and their dedicated owners. Our study sought to uncover the profound repercussions that the postictal phase can have on the QoL, not only for the affected dogs but also for their dedicated owners. Recognising the emotional and practical challenges that arise during this postseizure period is essential for tailoring holistic care plans that address the complete spectrum of epilepsy’s impact. Highlighting the significance of the postictal phase in the overall management of seizure disorders serves as a reminder that effective epilepsy management extends beyond just seizure control; it encompasses comprehensive care during the preictal, ictal, and postictal phases. Finally, our study aimed to explore potential therapeutic options for managing postictal signs. By identifying novel approaches and interventions, we aimed to enhance the overall well-being of dogs with epilepsy and alleviate the distress experienced during the postictal phase. This research represents a step towards more holistic and compassionate care for both canine patients and their devoted owners, ultimately improving their QoL and strengthening the human–animal bond.

Based on our gathered and analysed results, it is abundantly clear that the postictal phase exerts an influence on the QoL for both dogs and their owners, and it is virtually ubiquitous in occurrence. In the majority of cases, the postictal phase lasts up to 60 min, with abnormal behaviour such as disorientation, compulsive walking, and ataxia particularly affecting the QoL of dogs and owners. Resting in a darker environment as well as physical contact between owner and dog were the most common owner-applied measures. These owner-applied measures were reported to have a positive influence on managing the postictal signs, as perceived by the owners themselves. In contrast, the administration of ASM did not appear to have a substantial effect on improving the postictal signs, as reported by the respondents.

Just as the postictal phase is described as “the worst” in human medical studies [13], in this study, the majority of the respondents also expressed that postictal signs were severe. It is noteworthy that despite this perceived burden, the postictal phase remains significantly under-researched in both veterinary and human medicine. The pathophysiological mechanisms underlying the postictal clinical signs and the influence of these signs on the QoL are only vaguely reported in humans [8,26,27]. In veterinary medicine, there are no thorough reports specifically focusing on the postictal signs of dogs with epilepsy. The present study provides, firstly, insights drawn from the perspectives of dog owners into the intricacies of the postictal phase and its far-reaching effects on the daily lives of both dogs with epilepsy and their owners. Secondly, this research lays a solid foundation for future investigations aimed at enhancing the management of the postictal phase and, by extension, the QoL for dogs and their owners.

As mentioned in the Introduction, the postictal phase in humans is described as a transient state of the brain following a seizure, characterised by neurological deficits or psychiatric symptoms, often accompanied by EEG changes, and lasting for minutes to days [7]. It involves reduced neuronal excitability due to inhibitory signals and ion channels, with secondary changes that may impair brain function [8]. Apart from the ictus, secondary changes might also lead to brain dysfunction. According to a rodent study, severe postictal hypoxia occurs, reducing blood flow and oxygen supply to critical brain areas after a seizure [9].

The brain is organised in such a way that certain areas are responsible for certain tasks [9]. The clinical signs associated with an epileptic seizure are associated with the cerebrum and the thalamus [28]. The cerebrum is responsible for conscious perception and the processing of neuronal impulses; in the cerebral cortex, the various sensory inputs are combined and interpreted alongside each other [29]. Considering this information, the most common clinical signs such as compulsive walking and disorientation are conclusive. 

In addition to psychoses or lethargy, the most common symptoms in human medicine are headaches, coughing, automatisms, paresis, and aphasia. However, the fact that these clinical signs, with the exception of coughing, ataxia, and paresis, cannot be objectively assessed and reported in dogs by their owners does not preclude the possibility that similar symptoms may manifest in canine patients. The fact that dogs cannot communicate their symptoms in an effective or understandable manner to humans is a challenge to veterinary medicine; such a challenge is inevitably inherent in the research approach. Dogs seeking the proximity of their owner during the postictal phase might indicate that the affected animals feel discomfort. In this respect, this sign could be analogous to the findings in human medicine. Standardised criteria for assessing the clinical signs or the frequency of postictal signs are not provided to dog owners; therefore, subjective perception should not be ignored. 

Since this survey focused on the impact of the clinical signs in the postictal phase on the QoL of the dogs and their owners, the first element of interest was the duration of the clinical signs. Approximately half of the respondents (51%) stated that the clinical signs would last between 1 and 30 min, followed by 20% (57/282) of the owners who reported a period between 31 and 60 min. The survey results suggest that the duration of the postictal phase has an impact on the quality of life from the perspective of both owners and their dogs. For instance, with a postictal phase lasting under 30 min, only 3% of the respondents indicated that the postictal phase reached the highest level of negative influence on their dogs’ quality of life. When the postictal phase lasted between 31 and 60 min, this percentage remained at 3%. If the postictal phase lasted from 61 to 90 min, 7% of the respondents reported that the highest level of negative impact on their dogs’ quality of life was reached. This trend was even more pronounced when considering the owners’ quality of life. In cases where the postictal phase was under 30 min, 3% of the respondents stated that the postictal phase reached the highest level of negative influence. When the postictal phase lasted between 31 and 60 min, this percentage increased to 7%, and with a duration of 61 to 90 min, it increased to 13%.

According to the survey findings, it is possible that not all postictal signs were uniformly present or had an equivalent impact on the QoL of the affected dogs and their owner. Disorientation and repetitive pacing were identified as the primary and most frequently documented clinical indicators, emphasising their importance during the postictal phase. Following closely behind were ataxia and temporary blindness, contributing to the diversity of postictal manifestations observed in this canine population. Another intriguing investigative approach in this regard would be to examine the correlation between seizure type or epilepsy diagnosis and the most prevalent postictal symptoms. However, such an investigation would require a more targeted survey and would deviate from the intended purpose of this survey study. In our survey, the majority of the population consisted of dogs with idiopathic epilepsy and generalised seizures. However, we only included cases of idiopathic epilepsy in our analysis. Even though these correlations could not be assessed in this survey, it is likely that the postictal semiology is affected by both the epilepsy diagnosis and seizure type. More than 40% reported that, among the various postictal signs, disorientation or compulsive walking affected the QoL the most. The actual significance of a clinical sign for the affected dog is measured on the basis of the subjective evaluation by the owners. Since the time required to answer the questionnaire should remain short in order to generate the highest possible number of evaluable responses, it was decided not to establish uniform standards. Nevertheless, this approach appears to be reasonable and comprehensible. Despite these findings, other clinical signs should not be disregarded, as about 11% of the respondents stated that all clinical signs had an equal influence on the QoL of both the dog and its owner. This minority perspective underscores the importance of considering the holistic impact of various postictal signs, even if some may be more prevalent or conspicuous, as every dog’s experience with epilepsy can be unique.

In human clinical studies, it is stated that despite the frequent postictal complaints of patients, e.g., headaches, a common clinical practice by physicians is focusing only on the treatment of seizures. Consequently, the management of postictal signs, which can significantly impact a patient’s QoL, is frequently overlooked or given less attention [30]. This could be due to the fact that despite the important role of the postictal phase in the QoL, there are no evidence-based treatment protocols in human or veterinary medicine, and the evidence focusing on the postictal phase semiology and management is scarce [8,31]. In parallel, the so-far underestimated relevance of the postictal phase could well be a reason for the comparatively low level of research in this area. Therefore, there is still a lot of demand for research on the “after-effects” of an epileptic seizure.

Xu et al. conducted an experiment on rats to investigate the potential improvement in postictal symptoms through the application of deep brain stimulation. This innovative study aimed to shed light on promising therapeutic possibilities within the realm of epilepsy management. It is worth noting that, in EEG analysis, it is well established that the postictal phase is characterised by the presence of slow waves occurring in the frontal cerebral cortex [26]. The theory behind deep brain stimulation in the aforementioned rat experiment is based on the prevention of the postictal cortical slow wave activity. This type of activity, while prevalent during the postictal phase, can also manifest in other states of consciousness disorders, making it a critical target for investigation. The results of this study provided compelling evidence of the effectiveness of deep brain stimulation in achieving its intended objective. Notably, they indicated a substantial reduction in cortical slow wave activity during the postictal period, suggesting that this intervention may hold promise as a means to mitigate the neurological disruptions associated with epilepsy [26]. 

Whether neurostimulation is suitable for the treatment of postictal symptoms in the home setting is debatable. However, it is clear that further research in this regard can be worthwhile in order to achieve a possible improvement in the QoL. Neurostimulation in dogs such as vagus nerve stimulation [32,33,34,35,36], deep brain stimulation [37], and repetitive transcranial magnetic stimulation (rTMS) [38] can reduce the seizure frequency and severity. However, these studies did not specifically focus on the effect of neurostimulation on the postictal semiology and treatment of the relevant signs. In addition, apart from vagus nerve stimulation, neurostimulation can only be primarily performed in hospital settings by experienced veterinary personnel rather than the owners at home. Therefore, other therapeutic measures should also be considered for treating postictal effects in dogs with idiopathic epilepsy. 

Another therapeutic approach involves the reduction in hypoperfusion and the resulting hypoxia. In an experimental study conducted in the field of human medicine, hypoxia was linked to changes in behaviour in rodents [8]. To treat the lack of oxygen and the resulting postictal symptoms, Farrell et al. used medication that effectively blocks the activity of cyclooxigenase-2 or L-type calcium channels, which play a role in neurovascular coupling and thus vasoconstriction. A vasodilator, nifedipine, also prevented behavioural changes in rodents [9]. In people with epilepsy, behavioural therapies such as progressive muscle relaxation and cognitive behavioural therapy are being investigated as adjunctive therapies. These therapies should aim to treat cognitive and/or psychiatric comorbidities, in addition to seizures [39].

When evaluating the question regarding the improvement in postictal clinical signs due to the long-term medications administered, no clear tendency could be identified. About half of the responders (52%) stated that these were effective. Subjectively, however, the owner survey showed that the administration of emergency medication would have no positive effect on the postictal phase (approx. 71%). Long-term medication is administered over a long period of time. Whether and to what extent long-term drugs have an effect on the postictal phase can only be tested by discontinuing them for such a long period of time until the concentration of the medication in the blood is below the level of effectiveness. However, this carries the risk that epileptic seizures might relapse in a dramatic manner, which is the reason why such an approach cannot be justified. In addition, when assessing the improvement in postictal clinical signs through the administration of certain rescue medications, an objective effect can only be determined by not administering them and monitoring whether the deterioration of postictal signs occurs. According to the survey, 77% of the owners reported that other measures seemed to have remarkably stronger effects than the administration of long-term or emergency medication. The most commonly mentioned measures were calming procedures, such as rest, physical proximity to the owner, and a calm and dark environment. Even though antiseizure medications did not seem to have a considerable effect on postictal signs, it is likely that the postictal phase may need to be treated differently. Further research on the development of specific pharmacological agents that might reduce postictal signs is warranted.

This study may pose limitations in the interpretation of the results. The study was based on a survey of owner responses; therefore, a subjective evaluation of the postictal phase by the owners might have influenced the results, especially since owners’ emotional responses could not be eliminated when responses were provided. Standardised and objective evaluation criteria were not provided to assess clinical signs. Beyond the clinically obvious signs of the postictal phase, signs such as psychosis or headaches reported in humans could not be assessed in this survey; hence, some postictal semiological factors and the impact of particular signs on the QoL might have been missed. It is also possible that a bias occurred due to a higher participation of severely affected owners in the survey. Lastly, a clear demarcation between the epileptic seizure, postictal phase, and interictal phase was not always evident and could not be reliably assessed by the owners, making an accurate assessment challenging.

## 5. Conclusions

This study provides crucial insight into understanding the postictal phase and its relationship to the current antiseizure therapies as well as the QoL of both dogs with epilepsy and their owners. Overall, the survey results unequivocally demonstrate that specific postictal signs are not only common but also exert a profound and undeniable impact on the QoL of both dogs and their dedicated owners. The frequency, severity, or duration of the postictal phase might also be affected by the animal’s responsiveness or resistance to ASM. Interestingly, it becomes evident that ASM, while crucial for managing the ictal phase, may often prove ineffective in mitigating the distressing aspects of the postictal phase, further underscoring the need for alternative nonpharmacological interventions.

This insight highlights the urgency and importance of continued research focused on refining our understanding of the postictal phase and developing effective strategies to manage it. Such research endeavours are indispensable in the ongoing quest to enhance the QoL of dogs grappling with seizure disorders and, by extension, the well-being of their owners. By delving deeper into this often-neglected aspect of epilepsy management, we can aspire to provide a more comprehensive and compassionate approach to care that addresses the entire spectrum of the condition’s impact.

## Figures and Tables

**Table 1:** General information regarding dogs’ disease characteristics, clinical status, and rescue medication and demographics.

Country	Germany 65% (190/292); UK 13% (37/292); USA 12% (35/292); Austria 1% (4/292); Belgium 1% (2/292); Other 8% (24/292)
Breed	Crossbreed 23% (67/292); Border Collie 8% (24/292); Labrador Retriever 8% (23/292); Australian Shepherd 7% (20/292); Golden Retriever 3% (9/292); Collie 3% (8/292); Belgian Shepherd 2% (5/292); German Shepherd 1% (3/292); English Springer Spaniel 1% (4/292); Other 44% (129/292)
Sex	Male-neutered 34% (98/292); Entire-male 27% (80/292); Female-neutered 27% (79/292); Entire-female 12% (35/292)
Age	5.8 years (mean age); 8 years (median); 0–16 (range)
Diagnostics	History, signalment, and minimal blood database; urine examination 100% (292/292; Tier I: 100%); Aforementioned examinations plus MRI or CT and CSF 10% (29/292; Tier II: 10%); Aforementioned examinations plus EEG 1.4% (4/292 Tier III: 1.4%)
Clinical manifestation of seizures	Generalised 75% (219/292); Focal with secondary generalisation 14% (41/292); Focal 11% (31/292); I do not know 0.5% (1/292)
Length of the most common type of seizures	1–2 min 56% (164/292) 3–5 min 20% (59/292) under 1 min 17% (51/292) more than 5 min 6% (18/292)
Approximate monthly number of seizures	1 = 40% (116/292); Between 1 and 5 = 47% (138/292); Between 6 and 10 = 6% (17/292); Between 11 and 20 = 1% (4/292); >20 = 1% (3/292); I do not know 4% (13/292)
Incidence of cluster seizures	Not monthly but more than once per year 31% (91/292); Never 28% (81/292); Once per month 13% (38/292); More than once per month 12% (34/292); Once per year 7% (21/292); I do not know/not applicable 9% (27/292)
Incidence of status epilepticus	Never 61% (179/292); Not monthly but more than once per year 11% (32/292); Once per year 8% (23/292); More than once per month 6% (17/292); I do not know/not applicable 11% (31/292); Once per month 3% (10/292)
Long-term medication	Phenobarbital 81% (236/292); Levetiracetam 35% (102/292); Potassium bromide 31% (91/292); Imepitoin 14% (42/292); Nothing 9% (25/292); Zonisamide 5% (16/292); Pregabalin 3% (8/292); Gabapentin 2% (5/292); Felbamate 1% (2/292)
Rescue medication	Yes 71% (207/292); No 24% (71/292); No answer/not applicable 5% (14/292)
Rescue medication product	Diazepam 67% (139/207); Midazolam 15% (32/207); Levetiracetam 8% (16/207); Other 10% (20/207)
Rescue medication route	Rectal 64% (133/207); Intranasal 14% (30/207); Oral 14% (29/207); Other 6% (12/207); Buccal 1% (2/207); Sublingual 0.5% (1/207)

**Table 2:** Reported effects of the administered medication on the postictal phase.

Positive effects of antiseizure medication on the postictal signs	Yes 52% (148/282); No 48% (134/282)
Antiseizure medications with the strongest effect on postictal signs	Phenobarbital 57% (85/148); Potassium bromide 21% (31/148); Levetiracetam 17% (25/148); Imepitoin 7% (10/148); Zonisamide 1% (2/148); Pregabalin 1% (1/148); Gabapentin 1% (1/148), Phenytoin 1% (1/148); Other 3% (4/148)
Positive effects of rescue medication on the postictal signs	No 71% (201/282); yes 29% (81/282)
Rescue medications with the strongest effect on postictal signs	Rectal diazepam 57% (46/81); Oral levetiracetam 20% (16/81); Intranasal midazolam 17% (14/81); Other 6% (5/81)

## Data Availability

The data presented in this study are available upon request from the corresponding author.

## Supplementary Materials

The following supporting information can be downloaded at: https://www.mdpi.com/article/10.3390/ani14010103/s1, File S1: Questionnaire on the impact of the postictal phase of dogs with seizure disorders on quality of life—owner perspective.
