# Supplementary material for: The Postictal Phase in Canine Idiopathic Epilepsy: Semiology, Management, and Impact on the Quality of Life from the Owners’ Perspective
Source: Animals (Basel). 2023 Dec 27;14(1):103. doi: 10.3390/ani14010103 (PMC10778387; doi:10.3390/ani14010103)
Supplement: Supplementary file 1 [file animals-14-00103-s001.zip › animals-2786196-supplementary.pdf]

Dear Sir or Madam,

My name is Charlotte Kähn and I am working as a doctoral student in the Department of Neurology at the Department of Small Animal and Medicine of the University of Veterinary Medicine Hannover under direct supervision of Dr. Marios Charalambous and Professor Dr. Holger Volk.

My doctoral thesis is about the postictal phase in dogs. Specifically, we would like to assess how postictal phase is affected by the seizures and the medication administered as well as how it affects the quality of life of both dogs and their owners. Such information will i) provide us with a better understanding on the impact of postictal phase on dogs with epilepsy, and ii) allow us to develop enhanced management protocols in canine epilepsy. An individualised treatment focusing not only on the managment of the seizures with antiseizure medications but also on addressing signs related postictal phase could improve the overall management and quality of life of dogs with epilepsy.

Therefore, we urgently need your support. I am very grateful that you are taking the time to complete my survey. It will take you approximatly 10-15 minutes of your time. Your answers will be automatically scored by the LimeSurvey program and the results will be categorised manually based on the scores obtained. If you have any questions regarding the contact, please do not hesitate to contact me by email or phone.

Thank you,

Charlotte Kähn

If you would like to pause this survey an continue at a later time, please click " Load unfinished survey" in the upper right corner of the browser window.

To continue, please first accept our seurvey data policy by ticking the box.

Section A: A. General information about you and your dog

A1. In which country do you and your dog reside?

If you choose "Other country" please also specify your choice in the accompanying text field.

- Germany ☐
- Belgium ☐
- Netherlands ☐
- Italy ☐
- UK ☐
- USA ☐
- Austria ☐
- Other country ☐

Other country

[ ]

A2. What is your dog's breed?

If you choose 'Other breed' please also specify your choice in the accompanying text field.

- [ ] Cross-Breed
- [ ] Australia Shephard
- [ ] Belgian Shepherd
- [ ] Beagle
- [ ] Bernese Mountain Dog
- [ ] Border Collie
- [ ] Collie
- [ ] Border Terrier
- [ ] Cavalier King Charles
- [ ] English Springer Spaniel
- [ ] Cane Corso
- [ ] Dalmatian
- [ ] German Shepherd
- [ ] Italian Spinone
- [ ] Poodle
- [ ] Golden Retriever
- [ ] Labrador Retriever
- [ ] Shetland Sheepdog
- [ ] Finnish Spitz
- [ ] Magyar Viszlar
- [ ] Irish Wolfhound
- [ ] Petit Baset Griffon Vendeen
- [ ] Lagotto Romagnolo
- [ ] Other breed

Other breed

A3. What is the sex of your dog?

- Male ☐
- Male neutered ☐
- Female ☐
- Female neutered ☐

A4. How old is your dog (in full years)?

If your dog is under 1 year, please write 0.

☐☐

A5. What is the diet of your dog?

More than one choice may be applicable.

If you feed another food, please specify the name and brand of the commercial or clinical diet you give.

- Commercial wet food ☐
- Commercial dry food ☐
- Home-made cooked diet ☐
- Home-made raw diet ☐
- Specific clinical diet for epilepsy (e.g. Purina Neurocare) ☐
- Cannabidiol-Oil ☐
- Medium chain triglycerides-Oil ☐
- Vitamins ☐
- Other diet ☐

Other diet

[ ]

Section B: B. Information about the long-term (chronic) epileptic disorder of your dog

B1. Does your dog suffer from epilepsy or has history of recurrent seizures in the past?

- Yes ☐
- No ☐
- I do not know ☐

B2. Age of your dog when the first seizure occurred?

If your dog did not suffer from epilepsy or has history of recurrent seizures in the past, please tick "Not applicable".

- under 2 ☐
- Between 2 and 4 ☐
- Between 4 and 6 ☐
- Between 6 and 8 ☐
- Between 8 and 10 ☐
- Above 10 ☐
- I do not know ☐
- Not applicable ☐

B3. How was the diagnosis of epilepsy made?

If your dog did not suffer from epilepsy or has history of recurrent seizures in the past, please tick "Not applicable".

- History ☐
- Blood tests ☐
- Urine tests ☐
- MRI scan of the brain ☐
- CT scan of the brain ☐
- CSF analysis ☐
- EEG ☐
- I do not know ☐
- Not applicable ☐

B4. What was the (presumed or confirmed) diagnosis for your dog's seizure disorder?

If your dog did not suffer from epilepsy or has history of recurrent seizures in the past, please tick "Not applicable".

- Idiopathic epilepsy (i.e. no cause found, suspected genetic cause) ☐
- Structural epilepsy (i.e., intracranial pathology e.g., brain tumour, inflammation etc.) ☐
- Reactive seizures (i.e., metabolic diseases such as liver dysfunction, or intoxication) ☐
- I do not know ☐
- Not applicable ☐

B5. What is the clinical manifestation of the seizures in your dog?

If your dog did not suffer from epilepsy or has history of recurrent seizures in the past, please tick "Not applicable".

- [ ] Generalised (involving the whole body and limbs, loss of consciousness)
- [ ] Focal (involving only a part of the body, e.g., muscle contraction/twitching of face or limbs(s), increased salivation, licking, altered consciousness)
- [ ] Starting as focal then progressing to generalised
- [ ] I do not know
- [ ] Not applicable

B6. When you time the seizure with a watch, how long does your dog's most common type of seizures last on average? Record only the time of muscle twitching and not the recovery time.

If your dog did not suffer from epilepsy or has history of recurrent seizures in the past, please tick "Not applicable".

- [ ] Under 1 minute
- [ ] 1-2 minutes
- [ ] 3-5 minutes
- [ ] More than 5 minutes
- [ ] Not applicable

B7. What is the approximate monthly number of seizures your dog experiences?

If your dog has less than one seizure per month, please write "0,5".

If you don't know, please write the "99".

If your dog did not suffer from epilepsy or has history of recurrent seizures in the past, please write "100".

B8. How often does your dog experience cluster of seizures (cluster episode is defined as more than one seizures occuring all together within a 24-hour period)?

If your dog did not suffer from epilepsy or has history of recurrent seizures in the past, please tick "Not applicable".

- [ ] More than one cluster of seizures per month
- [ ] Once per month
- [ ] Not monthly but more than once per year
- [ ] Once per year
- [ ] Never
- [ ] I do not know
- [ ] Not applicable

B9. How often does your dog experience status epilepticus (defined as a continuous seizure activity, e.g., generalized muscle twitching or movements of face and limbs, lasting more than 5 minutes)?

If your dog did not suffer from epilepsy or has history of recurrent seizures in the past, please tick "Not applicable".

- More than once per month ☐
- Once per month ☐
- Not monthly but more than once per year ☐
- Once per year ☐
- Never ☐
- I do not know ☐
- Not applicable ☐

B10. Does your dog currently receive any medication to treat their epilepsy?

If your dog did not suffer from epilepsy or has history of recurrent seizures in the past, please tick "Not applicable".

- Yes ☐
- No ☐
- Not applicable ☐

B11. Please choose the antiseizure medication currently used for your dog's epilepsy?

If you choose 'Other medication' please also specify your choice in the accompanying text field.

If your dog did not suffer from epilepsy or has history of recurrent seizures in the past, please write "Not applicable".

- Phenobarbital (Epiphen, Phenoleptil) ☐
- Potassium bromide (Epilease, Libromide) ☐
- Imepitoin (Pexion) ☐
- Levetiracetam (Keppra) ☐
- Zonisamide ☐
- Gabapentin ☐
- Pregabalin ☐
- Felbamate ☐
- Phenytoin ☐
- Not applicable ☐

Other medication

Other medication

B12. What long-term antiseizure medication dosages (mg/kg) does your dog currently receive to manage epilepsy?

If your dog did not suffer from epilepsy or has history of recurrent seizures in the past, please write "Not applicable".

Write down the name, dosage and frequency of administration of every medication.

B13. Do you consult with your veterinarian for controlling and evaluation of the dosages and/or blood levels of the long-term antiseizure medication?

If your dog did not suffer from epilepsy or has history of recurrent seizures in the past, please write "Not applicable".

Every year as directed by my veterinarian

Last control was 2 years ago

Last control was more than 2 years ago

Never

Not applicable

B14. Do you use any rescue medication for acute emergency continuous (non-stop) seizures at home?

If your dog did not suffer from epilepsy or has history of status epilepticus in the past, please tick "Not applicable".

Yes

No

Not applicable

B15. What is the rescue medication you used to stop the continous seizure activity?

If you choose 'Other rescue medication' please also specify your choice in the accompanying text field.

Midazolam

Diazepam

Lorazepam

Levetiracetam

Other rescue medication

Other rescue medication

B16. Via which route do you administer the rescue medication?

If you choose 'Other route' please also specify your choice in the accompanying text field.

- Intranasal ☐
- Rectal ☐
- Buccal ☐
- Sublingual ☐
- Oral ☐
- Other route ☐

Other route

Section C: C. The postictal period

C1. After your dog stopped twitching or showed running movement the postictal period begins. What are the postictal signs (i.e., signs occurring after the seizure-related motor activity has stopped)?

If you choose "Other postictal signs" please also specify your choice in the accompanying text field.

- Disorientation ☐
- Repetitive vocalization ☐
- Vomiting ☐
- Compulsive walking ☐
- Urination and/or Pooping ☐
- Seeks closeness to the owner ☐
- Aggression ☐
- Blindness and falling into obstacles ☐
- Photophobia ☐
- Phonophobia ☐
- Ataxia (walking like drunk) ☐
- Lethargy and sleepiness ☐
- Altered degree of hunger an thirst ☐
- Coughing or spitting ☐

No postictal signs ☐

Other postictal signs ☐

Other postictal signs

C2. If you selected more than one postictal signs in question 22, which postictal sign is the most commonly present?

If you choose "Other postictal signs" please also specify your choice in the accompanying text field.

- Disorientation ☐
- Repetitive vocalization ☐
- Vomiting ☐
- Compulsive walking ☐
- Urination and/or Pooping ☐
- Seeks closeness to the owner ☐
- Aggression ☐
- Blindness and falling into obstacles ☐
- Photophobia ☐
- Phonophobia ☐
- Ataxia (walking like drunk) ☐
- Lethargy and sleepiness ☐
- Alteres degree of hunger and thirst ☐
- Coughing or spitting ☐
- All are equally present ☐
- Other postictal signs ☐

Other postictal signs

C3. If you selected more than one postictal sign in question 22, which postictal sign is the most severe and affects the most the QUALITY OF LIFE OF YOUR DOG?

If you choose "Other postictal signs" please also specify your choice in the accompanying text field.

- [ ] Disorientation
- [ ] Repetitive vocalization
- [ ] Vomiting
- [ ] Compulsive walking
- [ ] Urination and/or Pooping
- [ ] Seeks closeness to the owner
- [ ] Aggression
- [ ] Blindness and falling into obstacles
- [ ] Photophobia
- [ ] Phonophobia
- [ ] Ataxia (walking like drunk)
- [ ] Lethargy and sleepiness
- [ ] Altered degree of hunger and thirst
- [ ] Coughing or spitting
- [ ] All are equally severe
- [ ] Other postictal sign

Other postictal sign

C4. If you selected more than one postictal sign in question 22, which postictal sign is the most severe and affects the most YOUR OWN/PERSONAL QUALITY OF LIFE?

- Disorientation ☐
- Repetitive vocalization ☐
- Vomiting ☐
- Compulsive walking ☐
- Urination and/or Pooping ☐
- Seeks closeness to the owner ☐
- Aggression ☐
- Blindness and falling into obstacles ☐
- Photophobia ☐
- Phonophobia ☐
- Ataxia (walking like drunk) ☐
- Lethargy and sleepiness ☐
- Altered degree of hunger and thirst ☐
- Coughing or spitting ☐
- All are equally severe ☐
- Other postictal sign ☐

Other postictal sign

[ ]

C5. How often do you observe postictal signs following an epileptic seizure in your dog?

- Alway, occurring after every seizure (100%) ☐
- Often, occurring after more than half of the seizures (>50%) but not always ☐
- Sometimes, occurring after less than half of the seizures (<50%) ☐
- Rarely, occurring only a occasionally ☐

C6. How long in average do observe postictal phase in your dog in minutes (please time with a watch)?

If you don't know, please write the "99".

[| |]

C7. Using a scale from 1 (Not at all, not disturbing normal activities) to 5 (Could not be worse), indicate how much the postictal signs affect the normal activity and behavior of the dog?

	1-Not at all, not disturbing normal activities	2-Mild, slowing normal activities a little bit	3-Moderate, slowing normal activities severe	4-Severe, slowing normal activities very severe	5-Could not be worse, preventing normal activities
Effect of the postictal signs	☐	☐	☐	☐	☐

C8. Using a scale from 1 (Not at all severe) to 7 (Could not be worse), please indicate the severity of the postictal sign(s).

	1-Not at all severe	2-Very mild	3-Mild	4-Moder ate	5-Severe	6-Very severe	7-Could not be worse
Severity of the postictal signs	☐	☐	☐	☐	☐	☐	☐

C9. Using a scale from 1 (Not at all severe) to 7 (Could not be worse), please indicate the degree that the postictal sign(s) affect the overall quality of life of your dog.

	1-Not at all severe	2-Very mildly	3-Mildly	4-Moder ately	5-Severel y	6-Very severely	7-Could not be worse
Effect on the quality of life of the dog	☐	☐	☐	☐	☐	☐	☐

C10. Using a scale from 1 (Not at all severe) to 7 (Could not be worse), please indicate the degree that the postictal sign(s) affect the overall quality of life of yourself.

	1-Not at all severe	2-Very mildly	3- Mildly	4-Moder ately	5-Severel y	6- Very severely	7-Could not be worse
Effect on your own quality of life	☐	☐	☐	☐	☐	☐	☐

C11. Compared to the past (before starting any antiseizure medication), do you believe that the antiseizure medication you currently give to your dog has decreased the postictal signs (i.e., did you see improvement)?

Yes ☐

No ☐

C12. In your opinion, which antiseizure drug affected the postictal phase the most?

If you choose "Other antiseizure drug" please also specify your choice in the accompanying text field.

- Phenobarbital (Epiphen, Phenoleptil) ☐
- Potassium bromide (Epilease, Libromide) ☐
- Imepitoin (Pexion) ☐
- Levetiracetam (Keppra) ☐
- Zonisamide ☐
- Gabapentin ☐
- Pregabalin ☐
- Felbamate ☐
- Phenytoin ☐
- Other antiseizure drug ☐

Other antiseizure drug

[ ]

C13. Do you believe that the rescue antiseizure medication you currently give to your dog to manage the emergency seizures (status epilepticus or cluster seizures) has decreased the postictal signs (i.e., did you see improvement)?

- Yes ☐
- No ☐

C14. In your opinion, which rescue medication affected the postictal phase the most?

If you choose "Other rescue medication" please also specify your choice in the accompanying text field.

- Intranasal midazolam ☐
- Rectal diazepam ☐
- Oral levetiracetam ☐
- Other rescue medication ☐

Other rescue medication

[ ]

C15. Apart from the antiseizure medication, what other measures do you take to shorten/improve the postictal perios in your dog?

If you choose 'Other Measures' please also specify your choice in the accompanying text field.

- Oxygen ☐
- Rest ☐
- Physical closeness ☐
- Warming ☐
- Cooling ☐
- Darkness ☐
- Quiet ☐
- Medication ☐
- Nothing ☐
- Other measures ☐

Other measures

[ ]

C16. If you give medication to shorten/improve the postictal period in your dog, please indicate which.

Write down the name, dosage and type of administration of every medication.

[ ]

C17. Do you believe the measures you take to address the postictal signs are effective, i.e., crease or at least decrease the severity of the signs?

- Yes ☐
- No ☐

C18. Which measure do you believe has impactes the most the postictal phase?

If you choose "Other measures" please also specify your choice in the accompanying text field.

- Oxygen ☐
- Rest ☐
- Physical closeness ☐
- Warming ☐
- Cooling ☐
- Darkness ☐
- Quiet ☐
- Medication ☐
- Other measures ☐

Other measures

Thank you very much for your time an effort in answering the big questionnaire. Your data will be used only for the current study. The results will be published in a veterinary journal. Summaries of the results will be of course reported to you.

It would be great if you could support further epilepsy research. If you have filmed your dog`s epileptic seizures, please feel free to upload the videos usind the link below. The videos are purely for scientific purposes and to help better understanding and research the disease and seizure patterns in dogs to get closer to the goal of epilepsy control. For more information, please visit the website. Feel free to share the link. https://caninepilepsy.online/

Additionally, we would be very happy if our reach could be expanded. For this we need your help!

Please like our Facebook page (https://www.facebook.com/VetspecialistsAgainstEpilepsy)and our Instagram page (https://www.instagram.com/vetspecialistagainstepilepsy/)

The entire epilepsy team thanks you. We wish you and your dog health and much success in the treatment of epilepsy.
